# Candidate Drugs Screening for Behcet’s Disease Based on Bioinformatics Analysis and Mouse Experiments

**DOI:** 10.3389/fimmu.2022.895869

**Published:** 2022-06-21

**Authors:** Qinyun Xia, Chujun Lyu, Fang Li, Binbin Pang, Xiaoyu Guo, He Ren, Yiqiao Xing, Zhen Chen

**Affiliations:** ^1^ Eye Center, Renmin Hospital of Wuhan University, Wuhan, China; ^2^ School of Life Sciences and Biotechnology, Shanghai Jiao Tong University, Shanghai, China

**Keywords:** Behcet’s disease, potential drugs, EAU, rabeprazole, celastrol

## Abstract

**Background:**

Behcet’s disease (BD) is a chronic immune disease that involves multiple systems. As the pathogenesis of BD is not clear, and new treatments are needed, we used bioinformatics to identify potential drugs and validated them in mouse models.

**Methods:**

Behcet’s disease-related target genes and proteins were screened in the PubMed and UVEOGENE databases. The biological functions and pathways of the target genes were analyzed in detail by Gene Ontology (GO) and Kyoto Encyclopedia of Genes and Genomes (KEGG) analyses. A protein-protein interaction (PPI) network was constructed by the STRING database, and hub genes were identified by the Cytoscape plug-in CytoHubba. Gene-drug interactions were identified from the DGIdb database. Experimental autoimmune uveitis (EAU) mice were used as an animal model for drug validation.

**Results:**

A total of 249 target genes and proteins with significant differences in BD were screened, and the results of functional enrichment analysis suggested that these genes and proteins were more located on the cell membrane, involved in regulating the production of cytokines and affecting the activity of cytokines. They mainly regulated “Cytokine- Cytokine receptor interaction”, “Inflammatory bowel disease (IBD)” and “IL-17 signaling Pathway”. In addition, 10 hub genes were obtained through PPI network construction and CytoHubba analysis, among which the top 3 hub genes were closely related to BD. The DGIdb analysis enriched seven drugs acting together on the top 3 hub genes, four of which were confirmed for the treatment of BD or its complications. There is no evidence in the research to support the results in omeprazole, rabeprazole, and celastrol. However, animal experiments showed that rabeprazole and celastrol reduced anterior chamber inflammation and retinal inflammation in EAU mice.

**Conclusions:**

The functional analysis of genes and proteins related to BD, identification of hub genes, and validation of potential drugs provide new insights into the disease mechanism and potential for the treatment of BD.

## Introduction

Behcet’s disease (BD) is a chronic systemic disease involving multiple systems that generally presents with recurrent oral ulcers, genital ulcers, uveitis, vasculitis, skin lesions, and neurological and intestinal manifestations (1). BD is prevalent in Turkey, the Middle East, and East Asia (2, 3). However, the aetiology of BD is still unclear, and its diagnosis is complicated (4). Although occlusive vasculitis is a typical histopathological feature of BD, diagnosis still depends on typical clinical manifestations and clinical experience (5, 6).

Uveitis, one of the most common eye diseases in BD, is usually secondary to systemic manifestations, such as recurrent oral ulcers, after an average of 4 years (7). The typical characteristics of uveitis associated with BD are acute recurrent bilateral symmetrical or asymmetrical nongranulomatous panuveitis, accompanied by retinal vasculitis, and a tendency to self-heal (8). The treatment of BD mainly involves suppressing inflammation and reducing tissue damage. Although glucocorticoids and immunosuppressants are usually used for systemic anti-inflammatory treatment, treatment plans should be formulated based on clinical manifestations (9). Regarding the treatment of Behcet’s uveitis, in addition to glucocorticoids and immunosuppressants, other biological agents, such as infliximab (IFX) and adalimumab, have been proven to have certain therapeutic effects (10). Intravitreal injection of glucocorticoids is also an adjuvant treatment (11).

Currently, it is possible to control the progression of BD through the use of diverse therapeutic drugs and individualized treatments. However, it is also necessary to further understand the pathogenesis of this disease and introduce new, more effective drugs to control symptoms, improve prognosis, and even change the course of the disease (12). Here, bioinformatics was used to analyze genes or proteins most recently reported to be involved in BD to search for new potential drugs. Finally, we tested the possible impact of drug intervention on experimental autoimmune uveitis (EAU) mice, a model that has been widely used for human uveitis research (13). Overall, we aimed to provide new ideas for the treatment of BD.

## Material and Methods

### Identification of Behcet’s Disease-Related Target Genes and Proteins

Behcet’s disease-related target genes and proteins were obtained through the UVEOGENE database (http://www.uvogene.com) and PubMed (14) (15). We searched for papers related to BD from January 2016 to September 2021 in PubMed, and the relevant search terms included “Behcet’s disease”, “Neuro-Behcet’s disease”, “Bechet syndrome” and “Intestinal Behcet’s disease”. And detailed methods for screening target genes and proteins can be found in Section 1.1 of the **Appendix**.

### Target Enrichment Analysis

GO and KEGG analyses of target genes or proteins were performed using the Metascape database (http://metascape.org/) and the UniProt database (https://www.uniprot.org/) Detailed methods are present in Section 1.2 of the **Appendix**.

### Protein-Protein Interaction Network Analysis

A PPI network was constructed using the STRING database (https://string-db.org/) and Cytoscape software (version 3.8.2, California, USA). Detailed methods are present in Section 1.3 of the **Appendix**.

### Acquisition of Hub Gene

The top ten hub genes in the PPI network were enriched by Cytoscape plug-in cytoHubba. Detailed methods are present in Section 1.4 of the **Appendix**.

### Gene-Drug Interaction Analysis

Gene-drug interaction analysis can be achieved through the Drug Gene Interaction Database (DGIdb) version 4.2.0 (https://www.dgidb.org) (16). The drug-gene interaction network was constructed by the DGIdb database and Cytoscape Software. Detailed methods are present in Section 1.5 of the **Appendix**.

### Construction of Mouse Models of Simulated Ocular Behcet’s Disease

The Jackson Lab (Bar Harbor, ME, USA) provided B10.RIII mouse parents. Mice were bred and raised under specific pathogen-free (SPF) conditions. The EAU model was constructed by subcutaneous injection of 200 µl of emulsifier per mouse. The emulsifier was prepared by mixing 50 µg IRBP with an equal volume of CFA containing 1.0 mg/ml of Mycobacterium tuberculosis strain (MTB) (17). Our study was approved by the Ethics Committee of The Renmin Hospital of Wuhan University (WDRM20210708A). We made every effort to minimize the harm to the animals.

### Relative mRNA Expression of Hub Genes in EAU Models

TRIzol (Invitrogen, Carlsbad, CA, United States) was applied to extract total RNA from the retinas of EAU mice on the 14th day after injection. The extracted RNA was reverse transcribed into cDNA using the PrimeScript RT kit (Vazyme, China). SYBR premix (Vazyme, China) was used for final quantitative PCR detection. All the primers are listed in **Table 1**. The 2-^△△Ct^ cycle threshold method was applied to calculate the relative mRNA expression.

**Table 1:** Gene specific primer.

Gene	Primer sequence
**CCL2**	F: 5’-GCTACAAGAGGATCACCAGCAG-3’: R: 5’-GTCTGGACCCATTCCTTCTTGG-3’
**CSF2**	F: 5’-AACCTCCTGGATGACATGCCTG-3’ R: 5’-AAATTGCCCCGTAGACCCTGCT-3’
**IL-2**	F:5’- GCGGCATGTTCTGGATTTGACTC-3’ R:5’- CCACCACAGTTGCTGACTCATC-3’
**IL-13**	F:5’- AACGGCAGCATGGTATGGAGTG-3’ R:5’- TGGGTCCTGTAGATGGCATTGC-3’
**IL-4**	F:5’-ATCATCGGCATTTTGAACGAGGTC-3’ R:5’-ACCTTGGAAGCCCTACAGACGA-3’
**IFN-γ**	F:5’-CAGCAACAGCAAGGCGAAAAAGG-3’ R:5’-TTTCCGCTTCCTGAGGCTGGAT-3’
**IL-1β**	F:5’-TGGACCTTCCAGGATGAGGACA-3’ R:5’-GTTCATCTCGGAGCCTGTAGTG-3
**IL-17A**	F:5’-CAGACTACCTCAACCGTTCCAC-3’ R:5’-TCCAGCTTTCCCTCCGCATTGA-3
**TNF**	F:5’-GGTGCCTATGTCTCAGCCTCTT-3’ R:5’-GCCATAGAACTGATGAGAGGGAG-3
**IL-10**	F:5’-CGGGAAGACAATAACTGCACCC-3’ R:5’-CGGTTAGCAGTATGTTGTCCAGC-3'

### Drug Intervention Corresponding to Hub Genes in EAU Models

Celastrol purchased from MedChemExpress (MCE, HY-13067) was dissolved in DMSO (Sigma) at a concentration of 74 mM (18). Celastrol was aliquoted and stored at -20°C. EAU mice were intraperitoneally injected with celastrol at a dose of 1 mg/kg/day from Day 7 to Day 14, and control EAU mice were intraperitoneally injected with PBS containing the same concentration of DMSO (18). Rabeprazole (MCE, HY-B0656A) was dissolved in sterile PBS and intraperitoneally injected into EAU mice at a dose of 60 mg/kg/day from Day 7 to Day 14 (19, 20). Control mice were given an intraperitoneal injection with an equal volume of PBS. From Day 7 to Day 14 of EAU progression, ocular inflammation was observed and recorded daily under a slit-lamp microscope. On Day 14 of EAU, the eyeballs or retinas of mice were removed for HE staining and quantitative PCR. The quantitative PCR method is as mentioned above. The clinical score and histological score followed the scoring criteria proposed by Caspi RR (21).

### Statistical Analysis

All data were manifested as means ± standard error of the mean (Means ± SEM). Data were analyzed by GraphPad Prism 7.0(GraphPad Software, Inc, San Diego, CA, USA). The EAU score was processed by a Mann-Whitney U test. qRT-PCR experiments were performed by two-tailed Student’s t-test. P<0.05 was considered significant.

## Results

### Included Target Genes and Proteins Related to BD

**Figure 1:**
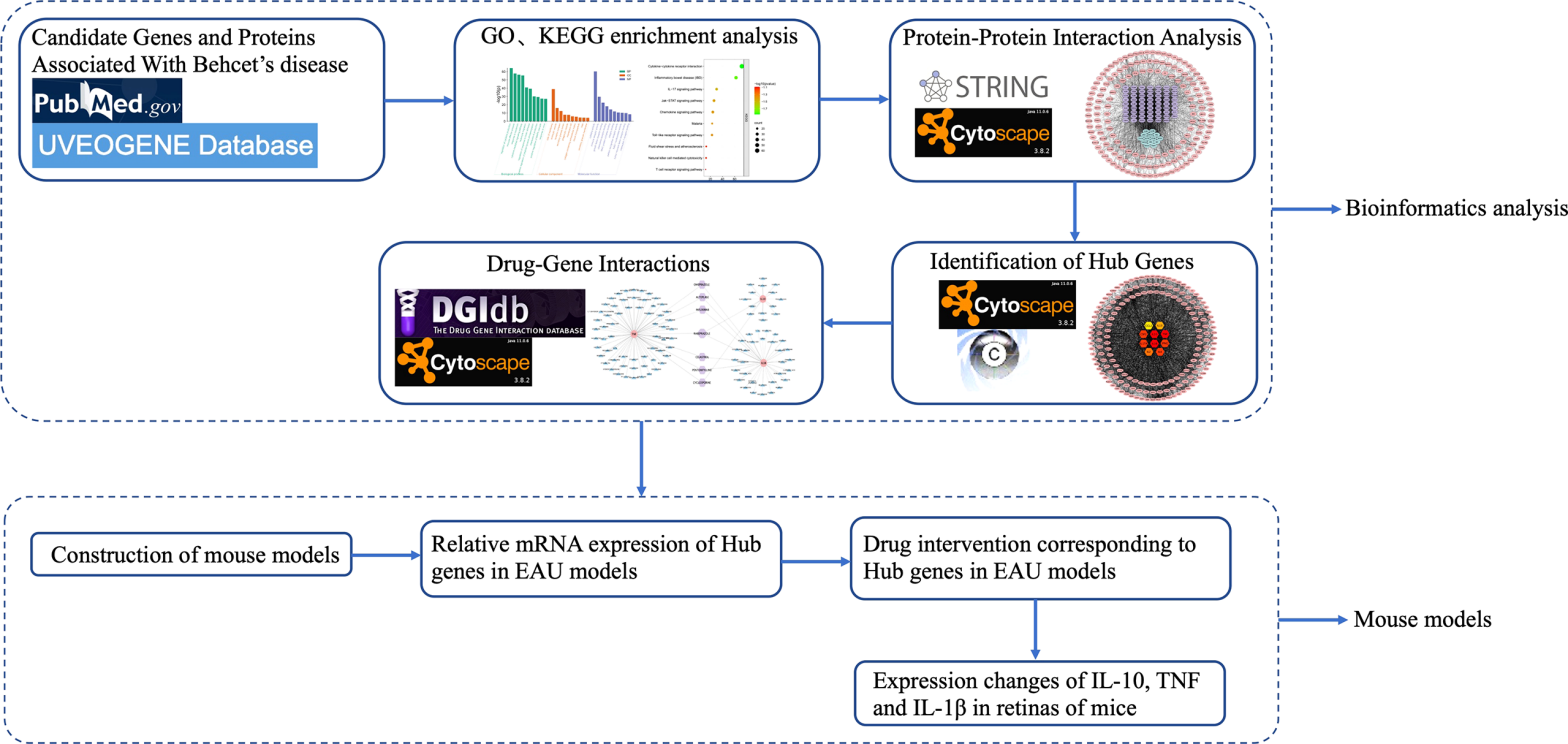
The flowchart of this study.

From January 2016 to September 2021, a total of 2654 articles were screened from PubMed, and 352 genes or proteins were selected according to the inclusion and exclusion criteria. After removing duplicates, a list of 249 differentially expressed genes or proteins was obtained that were significantly different from BD in the UVEOGENE database. Among them, 135 genes or proteins came from the UVEOGENE database and 114 from PubMed. The genes and proteins were normalized in the UniProt database, and the results are shown in **Supplementary Table 1**. The main research process of this study is shown in **Figure 1**.

### Enrichment Analysis of Target Genes and Proteins in BD

**Figure 2:**
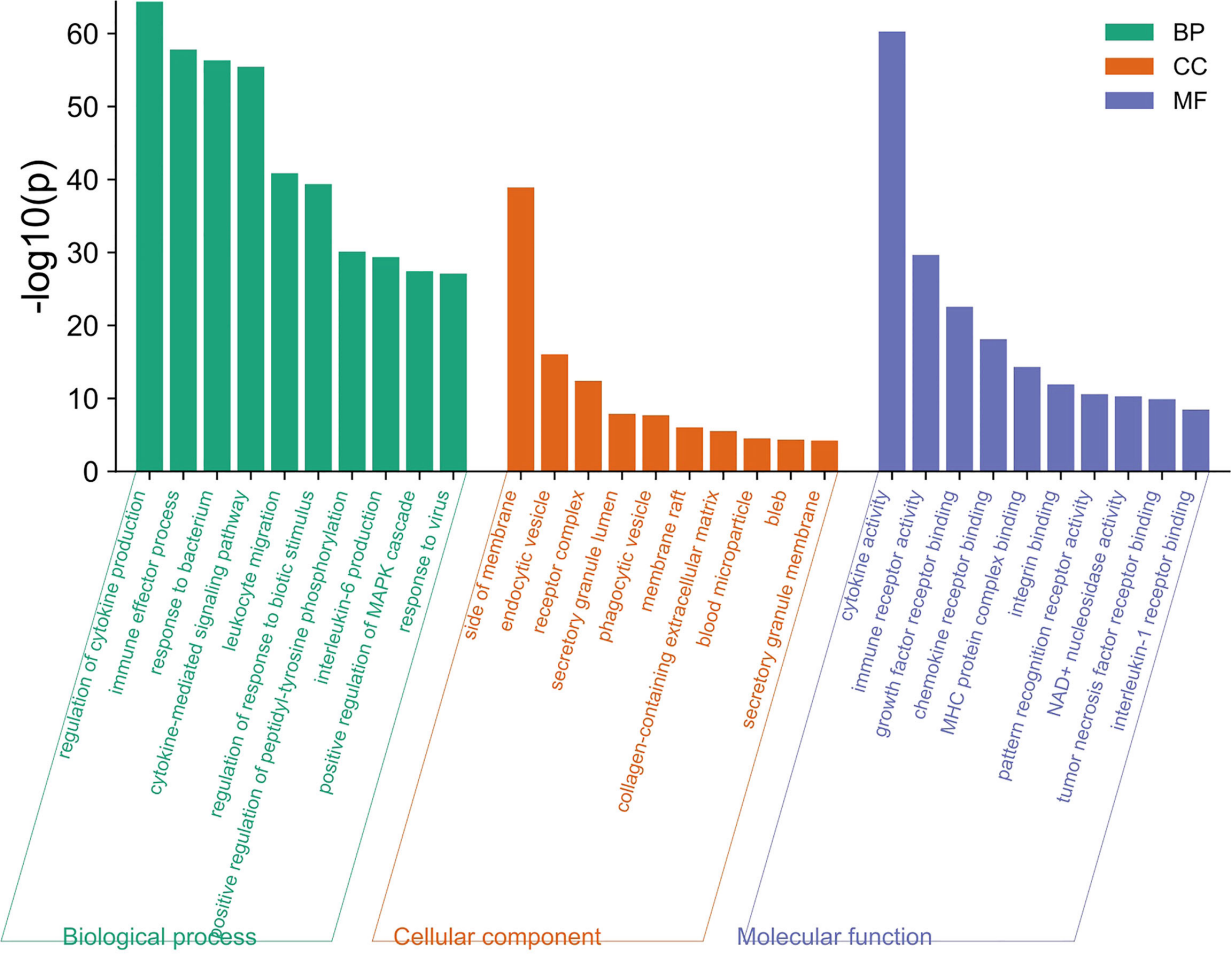
The top 10 items of GO enrichment analysis. The green, orange, and blue bars represent the analysis results of BP, CC, and MF, respectively. “Regulation of cytokine production”, “side of membrane”, “Cytokine activity” were the most significant difference in BP, CC, and MF respectively.

**Figure 3:**
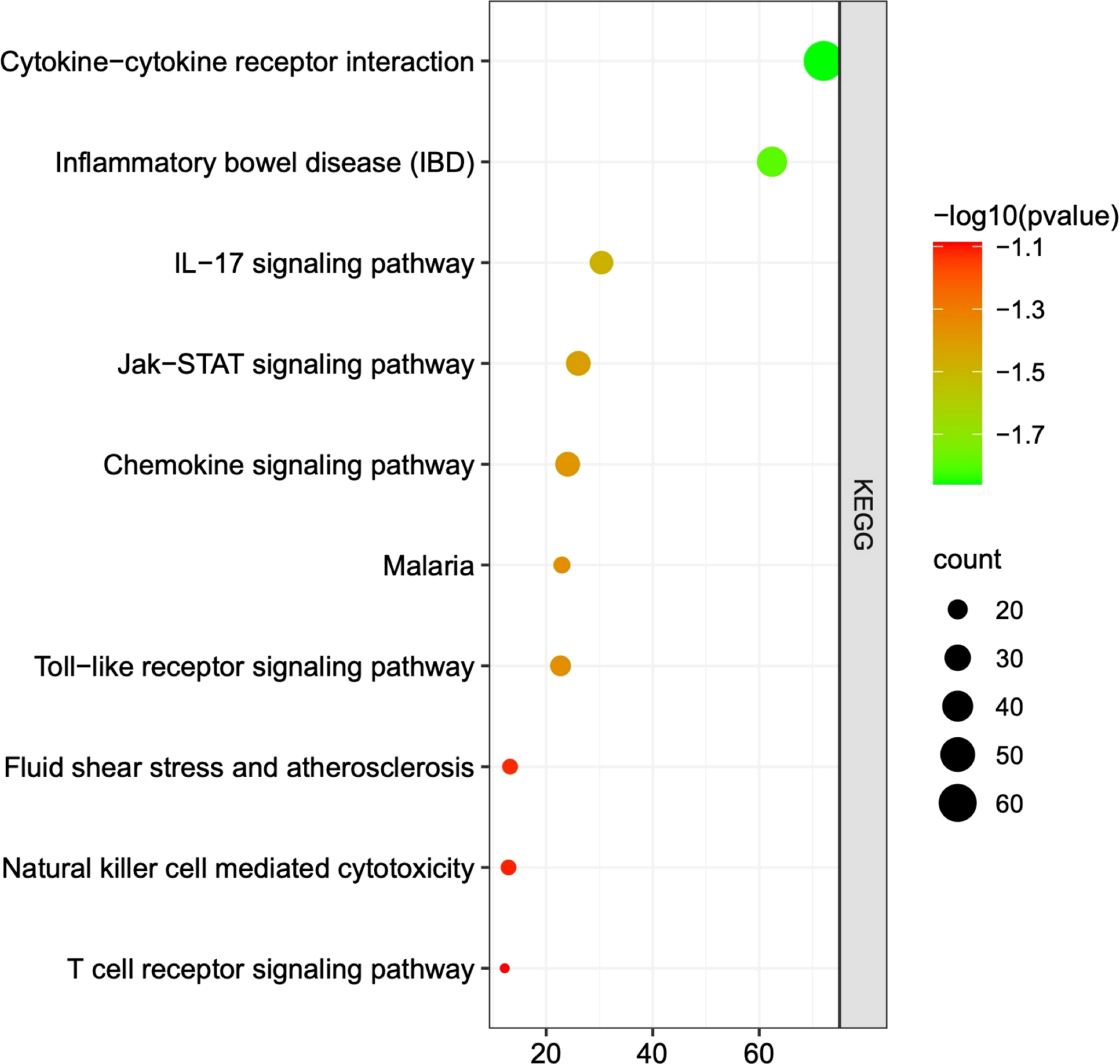
The top 10 pathways of KEGG analysis. Each dot represents a pathway, and the larger the dot is, the more genes it contains. The greener the dot is, the greater the difference. The three most significantly different pathways in KEGG analysis were “cytokine - cytokine receptor interaction”, “inflammatory bowel disease”, and “IL-17 signaling pathway”.

A total of 249 target genes and proteins were imported into the Metascape database for GO enrichment analysis and KEGG enrichment analysis. A total of 503 GO items were screened out in the Metascape database, including 337 BP items, 79 CC items, and 87 MF items, accounting for 67%, 15.7%, and 17.3%, respectively, and the top 10 items are shown in **Figure 2**. BP analysis showed that the included target genes and proteins primarily affected the “regulation of cytokine production”, “immune effector process”, “response to bacterium” and “cytokine-mediated signaling pathway”. In terms of cellular components (CC), these targets were mainly involved in the “side of membrane”, “endocytic vesicle” and “receptor complex”. In MF analysis, “cytokine activity”, “immune receptor activity” and “growth factor receptor binding” were affected. The KEGG enrichment analysis processed by the Metascape database screened 87 items (P<0.01) (**Supplementary Table 2**). The top 10 items are shown in **Figure 3**. The results suggested that these targets have a more significant impact on “Cytokine-cytokine receptor interaction”, “Inflammatory bowel disease (IBD) “and “ IL-17 signaling pathway” (**Figure 3**). In addition, 65 genes, 38 genes, and 25 genes were involved in these three pathways respectively. The diagram of the three signaling pathways is shown in **Supplementary Figures 1**–**3**.

### PPI Network Analysis and Hub Gene Recognition

**Figure 4:**
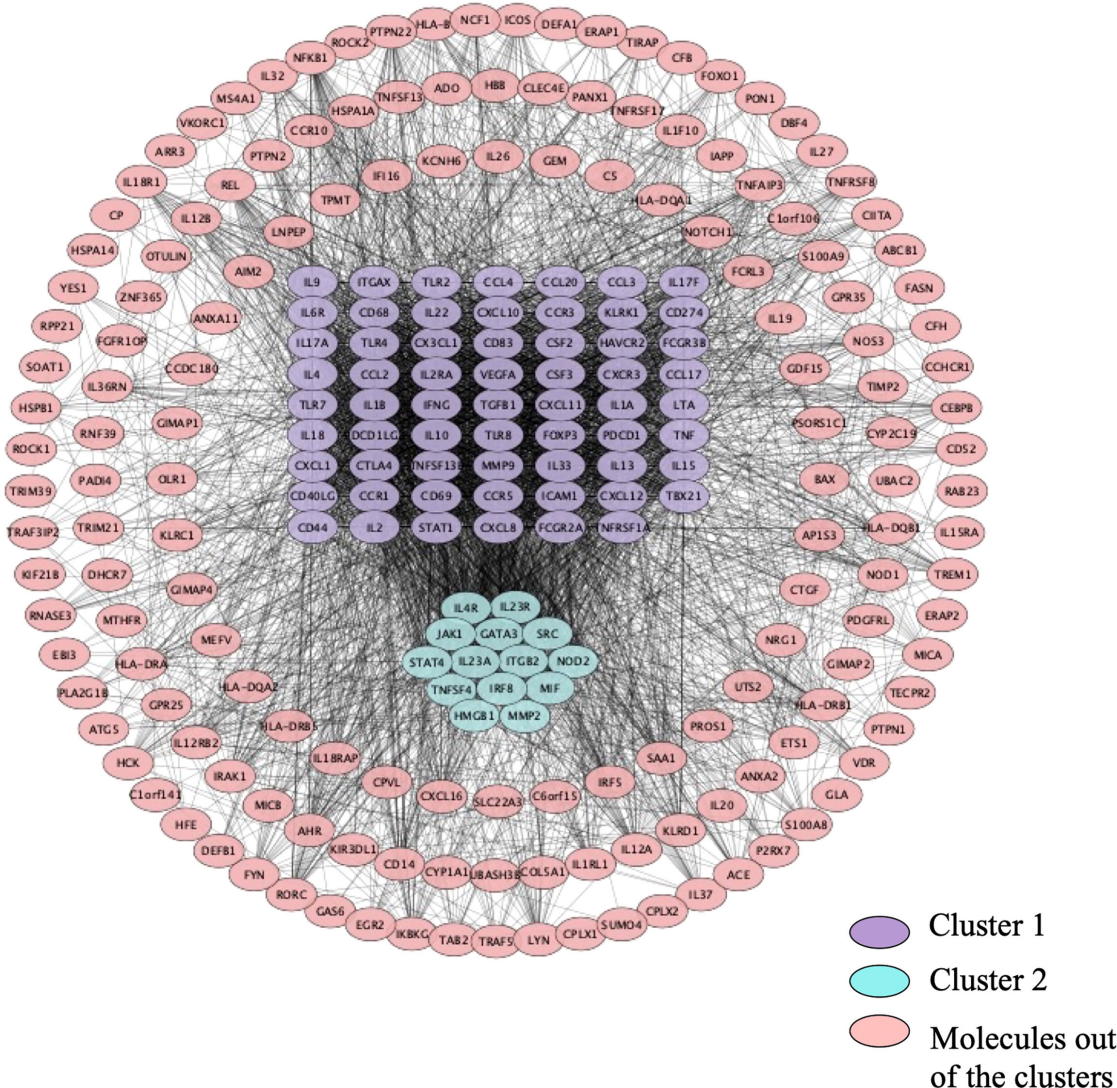
The PPI network. 246 nodes and 3946 edges were involved in the PPI network. Cluster 1 contains 62 nodes indicated in purple, and cluster 2 contains 14 nodes indicated in turquoise. Molecules in pink are not in any cluster.

**Figure 5:**
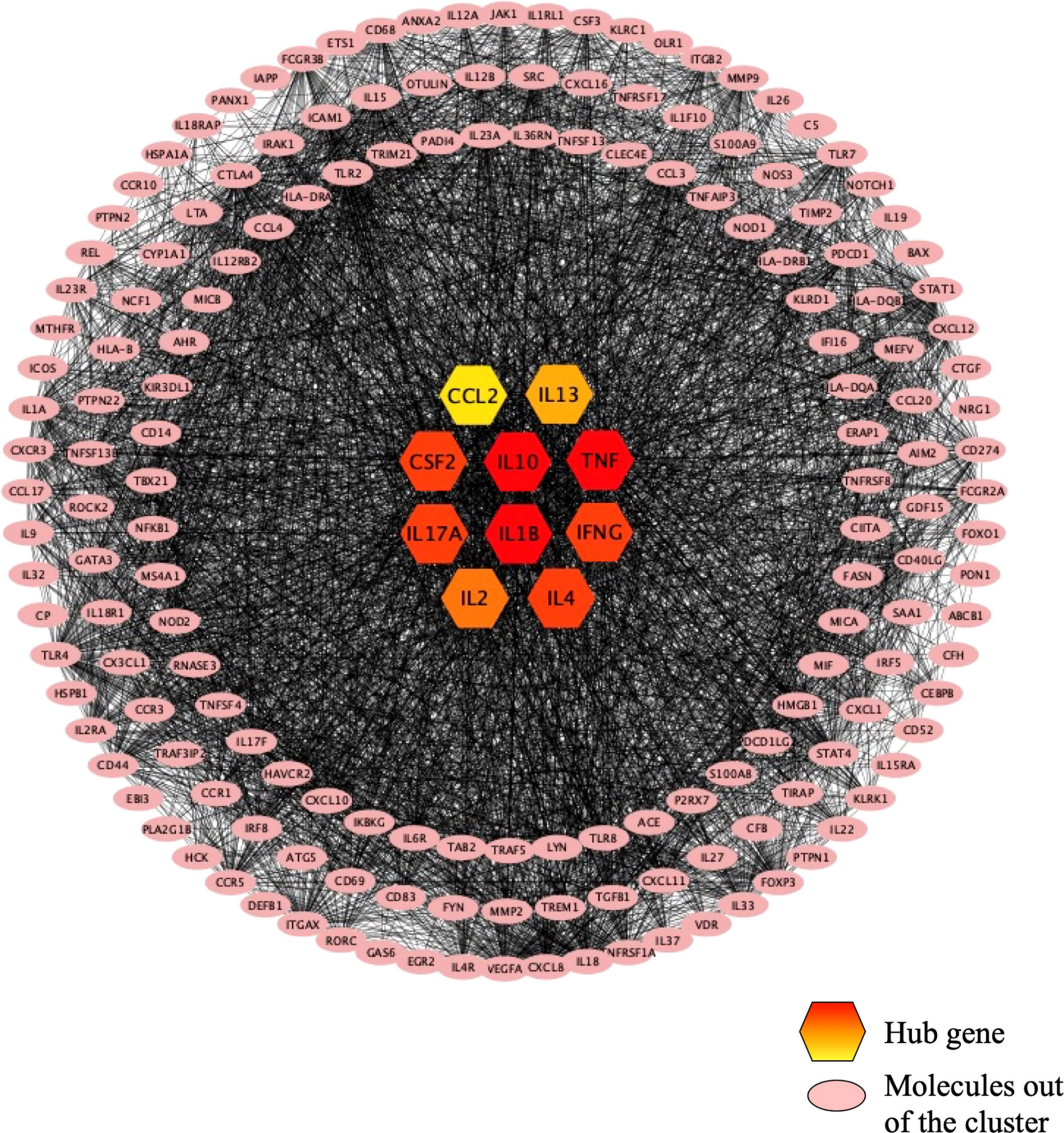
The top 10 hub genes recognized in the PPI network. The top 10 hub genes are in the center of the figure, and the color from red to yellow indicates the difference from large to small. TNF, IL1B, and IL10 are the top 3 hub genes. Molecules in pink are not in any cluster.

As shown in **Figure 4**, the PPI network was analyzed and constructed by the STRING database and Cytoscape software. In addition, 246 nodes and 3946 edges were involved in the PPI network. In addition, two clusters were obtained after processing and analysis by the MCODE plugin in Cytoscape software. Cluster 1containde 62 nodes and Cluster 2 contained 14 nodes (**Figure 4**). Moreover, the Cytohubba plugin was used to further analyze and process PPI network node signals. The MCC method was applied to select the top 10 hub genes with a score ≥5000 and node degree ≥10, including CCL2, IL-13, CSF2, IL-10, TNF, IL-17A, IL-1β, IFN-γ, IL-2, and IL-4 (**Figure 5**). The results calculated by the MCC method also suggested that TNF, IL-1β, and IL-10 were the top three hub genes respectively (**Figure 5**).

### Drug-Gene Interaction

**Figure 6:**
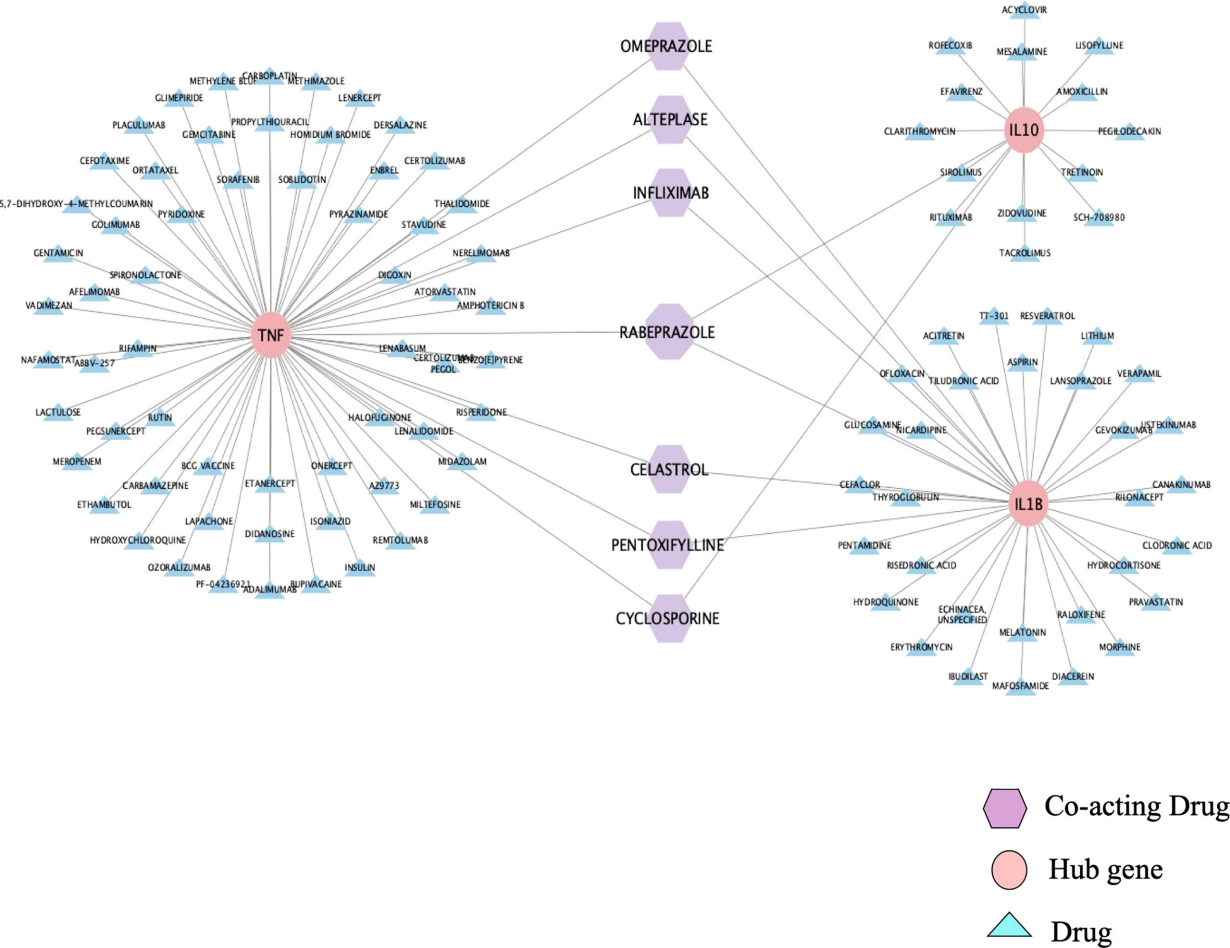
Drug-Gene Interaction. DGIdb database predicted six drugs acting on two hub genes and one drug acting on three hub genes. The pink represents the hub genes, the purple represents drugs that co-act on the hub genes, and the blue represents drugs that only act on one hub gene.

The DGIdb database was used to analyze the drugs interacting with the top three hub genes, and the results are shown in **Figure 6**. Among these drugs, 17 acted on IL-10, 37 acted on IL-1β, 68 acted on TNF, and 7 acted on any two or three genes simultaneously (**Figure 6**). All the results of the interaction with the first three hub genes are summarized in **Supplementary Table 3**, according to Interaction Type & Directionality, sources, Query Score, and Interaction Score. Among the seven drugs analyzed by the DGIdb database, infliximab, pentoxifylline, and cyclosporine have a clear therapeutic effect on BD. The usage, dosage, and side effects of these three drugs have been well studied (22). Alteplase, also known as recombinant human tissue plasminogen activator, rt-PA, is a thrombolytic agent primarily used for the treatment of Behcet’s disease-related thrombosis (23). However, the role of omeprazole, rabeprazole, and celastrol in BD has not been studied.

### Expression of Hub Genes in EAU

**Figure 7:**
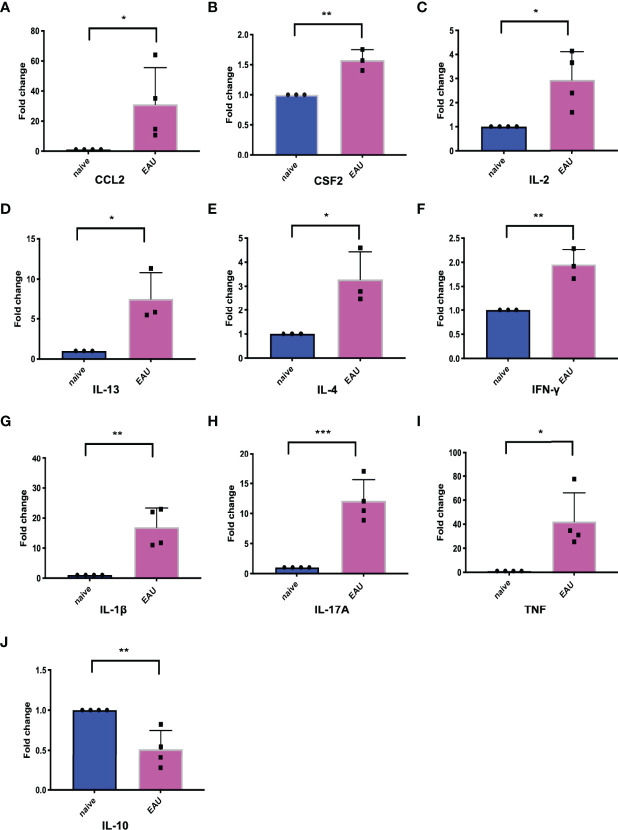
The expression of 10 hub genes in retinas of EAU mice and naive mice. The blue represents naive mice, and the red represents EAU mice. (A-J) represents the expression changes of CCL2, CSF2, IL-2, IL-13, IL-4, IFN-γ, IL-1β, IL-17A, TNF and IL-10, respectively. Except for the decreased expression of IL-10 in EAU, the other 9 hub genes were upregulated in EAU. *P < 0.05; **P < 0.01; ***P < 0.001 (two-tailed student’s *t*-test). Graphs show mean ± SEM.

Real-time PCR was applied to detect the expression of hub genes in the retinas of EAU mice. We compared the relative mRNA expression of hub genes in the retinas of naive and EAU mice on Day 14. The results showed that the relative mRNA expression of CCL2, CSF2, IL-2, IL-13, IL-4, IFN-γ, IL-1β, IL-17A, and TNF increased in the retina of EAU mice on Day 14, while IL-10 was decreased in the retina of EAU mice (**Figure 7**). CCL2, CSF2, IL-2, IL-13, IL-4, IFN-γ, IL-1β, IL-17A, TNF, and Il-10 showed statistically significant differences compared with naive mice (**Figure 7**).

### Celastrol Alleviated EAU

**Figure 8:**
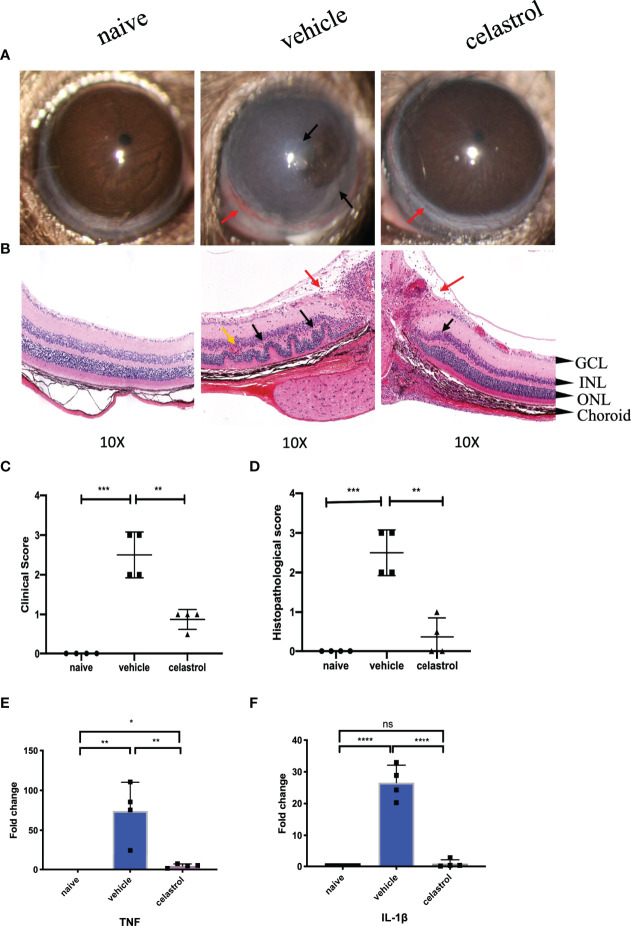
Celastrol alleviated EAU and inhibited the expression of TNF and IL-1β in the retinas of EAU. **(A)** Representative slit-lamp photographs of the naive group, the vehicle intervention group, and the celastrol intervention group on day 14 (n = 4/group). Red arrow, conjunctival hyperemia. Black arrow, inflammatory exudation. **(B)** Representative staining images of HE sections in the naive group, the vehicle intervention group, and the celastrol intervention group on day 14 (n = 4/group). Red arrow, vitreous inflammatory cell infiltration. Black arrow, retinal fold. Yellow arrow, neovascularization, and hemorrhage (GCL, ganglion cell layer; INL, inner nuclear layer; ONL, outer nuclear layer). **(C)** The clinical score of the naive group, the vehicle intervention group, and the celastrol intervention group on day 14 (n = 4/group). **(D)** The histopathological score of the naive group, the vehicle intervention group, and the celastrol intervention group (n=4/group). **(E)** qRT-PCR analysis of TNF in retinas of the naive group, the vehicle intervention group, and the celastrol intervention group on day 14 (n = 4/group). **(F)** qRT-PCR analysis of IL-1β in retinas of the naive group, the vehicle intervention group, and the celastrol intervention group on day 14 (n = 4/group). *P < 0.05; **P < 0.01; ***P < 0.001; ****P < 0.0001 (two-tailed student’s *t*-test). Graphs show mean ± SEM.

In EAU mice treated with celastrol, the inflammatory response of the anterior segment of the mouse was substantially reduced. Clear conjunctival hyperemia was still visible compared with naive mice (**Figure 8A**). A representative photograph of the anterior segment of the mouse was recorded on the 14th day, as shown in **Figure 8A**. Corneal edema and anterior chamber exudation were observed in the vehicle group, while other structures were not seen (**Figure 8A**). The clinical scores on Day 14 also showed that the scores of mice treated with celastrol were substantially lower than those of the vehicle group, and the difference was statistically significant (**Figure 8C**). In addition, the HE staining results also revealed that the EAU mice treated with celastrol had less structural damage to the retina on the 14th day and less inflammatory infiltration than the vehicle group, and there was a significant difference compared with the vehicle group (**Figures 8B–D**).

### Celastrol Inhibited the Expression of TNF and IL-1β in EAU the Retina of EAU Mouse Retinas

After 7 days of celastrol intervention, real-time PCR was used to detect the expression changes of TNF and IL-1β mRNA in the retina. Our results showed that TNF and IL-1β were upregulated in the retinas of mice in the vehicle group compared with those in the naive group, with significant statistical differences. However, TNF and IL-1β were significantly downregulated in the retinas of mice treated with celastrol, with significant differences from the vehicle group. The difference was statistically significant compared with the naive group (**Figures 8E, F**).

### Rabeprazole Alleviated EAU

**Figure 9:**
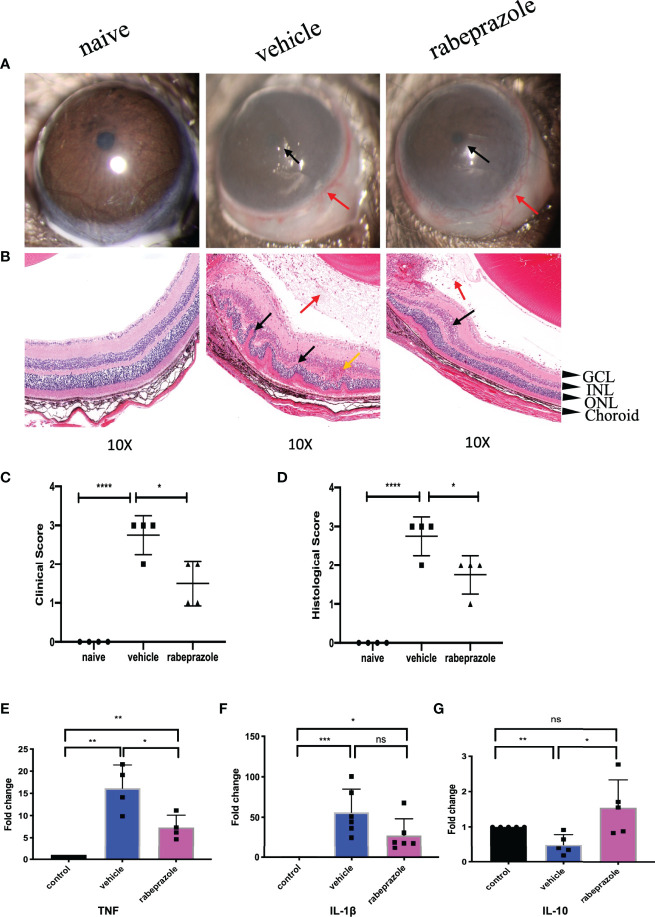
Rabeprazole alleviated EAU and inhibited TNF and IL-1β and promoted the expression of IL-10 in the retinas of EAU. **(A)** Representative slit-lamp photographs of the naive group, the vehicle intervention group, and the rabeprazole intervention group on day 14 (n = 4/group). Red arrow, conjunctival hyperemia. Black arrow, posterior synechiae, and inflammatory exudation. **(B)** Representative staining images of HE sections in the naive group, the vehicle intervention group, and the rabeprazole intervention group on day 14 (n = 4/group). Red arrow, vitreous inflammatory cell infiltration. Black arrow, retinal fold. Yellow arrow, neovascularization, and hemorrhage (GCL, ganglion cell layer; INL, inner nuclear layer; ONL, outer nuclear layer). **(C)** The clinical score of the naive group, the vehicle intervention group, and the rabeprazole intervention group on day 14 (n = 4/group). **(D)** The histopathological score of the naive group, the vehicle intervention group, and the rabeprazole intervention group (n = 4/group). **(E)** qRT-PCR analysis of TNF in retinas of the naive group, the vehicle intervention group, and the rabeprazole intervention group on day 14 (n = 4/group). **(F)** qRT-PCR analysis of IL-1β in retinas of the naive group, the vehicle intervention group, and the celastrol intervention group on day 14 (n = 6/group). **(G)** qRT-PCR analysis of IL-10 in retinas of the naive group, the vehicle intervention group and the rabeprazole intervention group on day 14 (n = 5/group) *P < 0.05; **P < 0.01; ***P < 0.001; ****P < 0.0001 (two-tailed student’s *t*-test). Graphs show mean ± SEM.

In the rabeprazole group on Day 14, the anterior images showed moderate iridocyclitis with clear pupils and obvious conjunctival hyperemia, while in the vehicle group, corneal edema was more serious with unclear pupils and obvious conjunctival hyperemia (**Figure 9A**). Clinical scores showed significant differences between the two groups (**Figure 9C**). Meanwhile, the HE staining results on Day 14 indicated that the retinal structure of mice treated with rabeprazole was clear, the damage was lighter than that of the vehicle group, and there were fewer inflammatory cells found in the vitreous than were found in that of the vehicle group. Histological scores also showed significant differences between the two groups (**Figures 9B–D**).

### Rabeprazole Inhibited TNF and IL-1β and Promoted the Expression of IL-10 in the Retinas of EAU Mice

To investigate whether rabeprazole affects the expression of the top 3 hub genes TNF, IL-1β, and IL-10 in the retina, we also extracted the retinas of EAU mice 7 days after rabeprazole intervention and measured the expression of these three genes by real-time PCR. They were compared with naive mice and vehicle mice. Our results showed that TNF expression increased in the retinas of mice on Day 14 after treatment with rabeprazole, which was significantly lower than that of the vehicle group, and there were significant differences between the naive group and vehicle group (**Figure 9E**). IL-1β expression was lower compared to that in the vehicle group, but there was no significant difference with the vehicle group (**Figure 9F**). Meanwhile, the expression of IL-10 in the retinas of the rabeprazole group was increased, and the difference was statistically significant compared with that in the vehicle group, but not statistically significant compared with that in the naive group (**Figure 9**).

## Discussion

BD is a refractory disease that affects patients worldwide (24). The treatment of BD remains extremely challenging, although it is possible to select appropriate treatment strategies for patients with specific phenotypes (25, 26). Advances in bioinformatics research provide new approaches for us to process the biological information for some diseases and predict possible therapeutic drugs. In this study, we analyzed the function and pathways of BD-related target genes through bioinformatics, and enriched hub genes to further explore potential investigational drugs.

Combining the results of the searches of the PubMed and UVEOGENE databases, we found 249 genes and proteins that were significantly different in BD. Subsequently, we performed GO and KEGG enrichment analyses on the 249 genes and proteins using the Metascape database. The results of the GO enrichment analyses indicated that these genes and proteins were closely related to “regulation of cytokine production”, “immune effector process “, “response to bacterium” and “cytokine activity”. Indeed, multiple studies of BD have confirmed that a large number of inflammatory cytokines are involved in BD, such as TNF-α, IL-6, IL-17A, and IL-10 (27–29). In addition, a pathogenic theory for BD suggests that it may be caused by the activation of the innate or adaptive immune system due to bacterial or viral infection or autoantigen damage, thus inducing the production of a large number of inflammatory cytokines and chemokines that participate in the immune-inflammatory response (2).

KEGG enrichment analyses showed that the 249 genes and proteins were mainly enriched in the Cytokine - Cytokine receptor interaction, IBD, and IL-17 signaling pathways. Currently, multiple cytokine-cytokine receptors interactions, such as IL-23 and IL-23R, TNF-α and TNFR1 or TNFR2, and IL-17A or Il-17F and IL-17RA or IL-17RC, have been confirmed in BD (2, 30, 31). As a member of the IL-12 family, IL-23 is an important cytokine that promotes Th17-cell differentiation (32). The binding of IL-23 to IL-23R can activate Th17 cells, promote the release of inflammatory cytokines, such as IL-17, IL-6, and TNF-α, and enhance the inflammatory response (33). In addition, activation of the IL-23/IL-17 pathway is involved in IBD and BD (29). Furthermore, our results showed that 25 target genes were enriched in the IL-17 pathway, which also suggests that the IL-17 pathway is important in BD. Moreover, identification of the IBD pathway also explained that the occurrence of immune inflammation and the activation of Th1, Th17, and Th2 cells were closely related to the activation of multiple pathways such as the Nod-like receptor signaling pathway, Toll-like receptor pathway, and Cytokine - Cytokine pathway.

To explore the connections among target genes or proteins, we constructed a protein-protein interaction network that divided target genes or proteins into three categories according to the interaction scores: one was clustered with a purple background, one was clustered with a green background, and the rest were placed outside the clusters. Subsequently, the Cytoscape plugin was applied to enrich 10 hub genes (CCL2, IL-13, CSF2, IL-10, TNF, IL-17A, IL-1β, IFN-γ, IL-2, IL4). Among the 10 hub genes, CCL2 was also named MCP1, and CSF2 was also named GM-CSF; nine of the hub genes, excluding CSF2, came from UVEOGENE databases. Furthermore, the enrichment results for the hub genes also suggested that TNF, IL-1β, and IL-10 are the most critical hub genes. TNF is a key pathogenic factor of BD, and some studies have shown that TNF is related to disease activity in BD (34) (35). TNF is highly expressed in various tissues in BD patients, such as oral ulcer tissue, the aqueous humour, and intestinal lesions (36–38). At present, there are many kinds of TNF-α blockers available for the treatment of BD, such as IFX, alemtuzumab (ADA), and etanercept (ETC) (39). Moreover, IFX and ADA have been recommended as first-line treatments for severe posterior uveitis associated with BD (40). IL-1β, a proinflammatory factor, is mainly secreted by monocytes, macrophages, and dendritic cells (DCs) and can induce the release of various inflammatory chemokines and promote the production of immune inflammation (25) IL-1β is highly expressed in the serum of patients with BD, and targeted inhibition of IL-1β has also been shown to be effective in the treatment of BD (41). Currently, canakinumab, gevokizumab, and anakinra, which are all IL-1 blockers, have been proven to be effective in the treatment of BD (42–44). IL-10 is an anti-inflammatory cytokine secreted by Th2 cells that mainly antagonizes proinflammatory factors, and promotes the activation of B cells to produce antibodies (45, 46). Moreover, three BD risk alleles rs1518111A, rs1800872A, and rs1800871T were related to IL-10 (47, 48).

To explore potential drugs, we analyzed the association between genes or drugs and the top 3 hub genes through the DGIdb database. Our results were centered on seven drugs, including IFX, pentoxifylline, and cyclosporine, which are clinically appropriate for the treatment of BD, and alteplase can be used for the treatment of BD complications (22, 23). However, the remaining three drugs have not been proven to be effective in the treatment of BD. Celastrol is a bioactive ingredient of *Tripterygium wilfordii*, which has been proven to have anti-inflammatory, antitumor, and antineovascularization effects in some studies (49, 50). Several studies have shown that celastrol has a certain anti-inflammatory effect on IBD, experimental autoimmune encephalomyelitis (EAE), and psoriasis models (51–54). In recent years, Chinese doctors have paid more attention to clinical experiments evaluating the use of celastrol in treating rheumatoid arthritis, idiopathic membranous nephropathy, and IBD (NCT01613079, NCT01161459, NCT02044952). Omeprazole and Rabeprazole are proton pump inhibitors (PPIs) that are currently mainly used for the treatment of gastrointestinal diseases, including gastroesophageal reflux disease, gastric and duodenal ulcers, and Zollinger-Ellison syndrome (55). Omeprazole is a first-generation PPI approved for clinical treatment, and it was followed by lansoprazole, rabeprazole, and other PPIs (56). Although PPIs are primarily used to treat digestive diseases, studies have shown that they may have anti-inflammatory effects in addition to inhibiting acid production (57). In recent years, PPIs have played an important role in inhibiting antigen presentation by inhibiting the expression of TNF-α and IL-1β induced by different pathogen-associated molecular patterns (PAMPs). In addition, PPIs inhibit the release of a large number of inflammatory factors caused by Toll-like receptor signaling activation in monocytes (58). Both omeprazole and rabeprazole have been reported to exert anti-inflammatory effects related to the inhibition of NF- κB activation (59–61). Furthermore, a number of studies have confirmed that PPIs can not only reduce inflammation in the human gastrointestinal epithelium but also reduce inflammation in the human lung epithelium and psoriasis (60, 62, 63). However, the treatment of other systemic inflammatory diseases with PPIs has rarely been reported.

Next, to verify whether these drugs might inhibit the inflammatory response in BD, we constructed an EAU mouse model with B10RIII mice (64). We compared the expression of hub genes in the retinas of EAU mice between a pre-modeling timepoint and Day 14 after modeling. The results showed that IL-10 expression was reduced in the retinas of EAU mice, but the expression of the other 9 hub genes was increased; these 10 hub genes significantly differed between the model and control mice. The data suggested that these 10 hub genes are involved in EAU and that EAU is a good research model for BD uveitis. Subsequently, we selected celastrol and rabeprazole from among the three unproven drugs to treat EAU mice. Although our study results pointed to both omeprazole and rabeprazole, considering that both drugs are metabolized by the CYP450 enzyme, we chose rabeprazole for experimental verification and found that rabeprazole had little effect on the enzyme CYP450 (65). In the EAU mouse model, EAU usually starts between Days 7 and 9, peaks on approximately Day 14, and then gradually resolves (17). We chose to administer treatment from the onset of EAU to the peak of the disease. Our results showed that after treatment with celastrol for 7 days, anterior chamber inflammation was significantly reduced, the retinal structure was slightly intact, and the retinal pro-inflammatory factors TNF and IL-1β were significantly downregulated, suggesting that celastrol could effectively alleviate EAU. In addition, in mice treated with rabeprazole, the inflammation in the anterior part of the mice was slightly reduced compared with that in vehicle-treated mice, and the retinal damage was less severe. The expression levels of the proinflammatory factors TNF and IL-1β in the retinas were lower in rabeprazole-treated mice than in vehicle-treated mice, while the expression of the anti-inflammatory factor IL-10 was upregulated compared with that in vehicle mice. These results indicated that rabeprazole can alleviate EAU to a certain extent.

In a short, target functional analysis and potential drug mining for BD highlighted the effectiveness of network pharmacological approaches. However, uveitis represented by the EAU model is just one of the typical manifestations of BD, so these agents need to be validated in many models. We also demonstrated the anti-inflammatory effect of rabeprazole in EAU for the first time, but the mechanism and side effects remained unclear. These results are still worthy of further study, which may provide some inspiration for the treatment of BD. In addition, the bioinformatics analysis may be exploratory, our study focused on PPIs, which are not clinically used as anti-inflammatory agents, it is also worth noting that an increasing number of studies have shown the anti-inflammatory properties of PPIs. Furthermore, our bioinformatic analysis focused on the existing data and the addition of future information that could affect our results. Despite these limitations, this study is the first preliminary and basic experimental verification of BD target gene enrichment-related drugs and provides new insights into the disease mechanisms and therapeutic potential of BD.

## Data Availability Statement

The datasets presented in this study can be found in online repositories. The names of the repository/repositories and accession number(s) can be found in the article/**Supplementary Material**.

## Ethics Statement

The animal study was reviewed and approved by the Ethics Committee of the Renmin Hospital of Wuhan University (WDRM20210708A).

## Author Contributions

The design and conception of the manuscript were completed by YX and ZC. Data collection and collation were performed by QX and CL. Bioinformatics analysis was finished by CL and FL. The EAU model constructed by QX, BP, and XG. Drug validation-related experiments were performed by QX, FL, and BP. Data collation and mapping were finished by CL, QX, and HR. Manuscript writing and revision were performed by QX, CL, FL, YX, and ZC. All authors contributed to the article and approved the submitted version.

## Funding

This study was supported by the Hubei health and family planning commission supporting projects WJ2017Z004 and WJ2021M156.

## Conflict of Interest

The authors declare that the research was conducted in the absence of any commercial or financial relationships that could be construed as a potential conflict of interest.

## Publisher’s Note

All claims expressed in this article are solely those of the authors and do not necessarily represent those of their affiliated organizations, or those of the publisher, the editors and the reviewers. Any product that may be evaluated in this article, or claim that may be made by its manufacturer, is not guaranteed or endorsed by the publisher.
