# Supplementary material for: Candidate Drugs Screening for Behcet’s Disease Based on Bioinformatics Analysis and Mouse Experiments
Source: Front Immunol. 2022 Jun 21;13:895869. doi: 10.3389/fimmu.2022.895869 (PMC9253297; doi:10.3389/fimmu.2022.895869)
Supplement: Supplementary file 1 [file DataSheet_1.docx]

**1 Material and Methods**

**1.1 Identification of Behcet’s disease-related target genes and proteins**

Behcet's disease-related target genes and proteins were obtained through a literature search and the UVEOGENE database (http://www.uvogene.com)^(1)^. PubMed was used as the literature search engine^(2)^. We searched for papers related to BD from January 2016 to September 2021 in PubMed, and the relevant search terms included “Behcet’s disease”, “Neuro-Behcet’s disease”, “Bechet syndrome” and “Intestinal Behcet’s disease”. The target genes and proteins are the following: (i) genes or proteins found to be significantly different between Behcet's patients and normal controls. (ii) significantly different genes and proteins in BD in the UVEOGENE database. Genes and proteins in the following categories were excluded: (i) genes and proteins with differences that were identified only in animal studies. (ii) genes or proteins associated with BD in articles or the UVEOGENE database, with no statistically significant difference compared with normal controls. All data collection and collation were performed independently by two researchers, and then the research team regularly discussed and resolved any misunderstandings or data discrepancies.

**1.2 Target enrichment analysis**

GO and KEGG analyses are two common methods for gene enrichment analysis. GO analysis, or Gene Ontology Analysis, primarily classifies the function of genes or proteins from three aspects: biological processes (BP), molecular functions (MF), and cellular components (CC). KEGG analysis is based on the Kyoto Encyclopedia of Genes and Genomes, which represents a set of genes in the genome in the form of interacting molecular networks^(3)^. GO and KEGG analyses of target genes or proteins were performed using the Metascape database (http://metascape.org/), which is an effective tool for multiomics-level enrichment analysis^(4)^. The gene names of the selected target genes and proteins were normalized in the UniProt database (HTTPS:// [www.uniprot.org/](http://www.uniprot.org/)). Then, the normalized target genes or proteins were imported into the Metascape database, and the species was restricted to humans. After database retrieval and transformation, the threshold P<0.01 was set for enrichment analysis of GO biological processes and KEGG signaling pathways.

**1.3 Protein-Protein Interaction network analysis**

The implementation of different functions in cells depends on the interaction between protein molecules^(5)^. A large number of technologies and algorithms exist for the construction of PPI networks, which can interpret protein-protein interactions in detail. A PPI network was constructed using the STRING database (https://string-db.org/) and Cytoscape software (version 3.8.2, California, USA). The normalized target genes and proteins were imported into the STRING database, and the protein interaction information was downloaded. Then the information was imported into Cytoscape 3.8.2 software and analyzed by the MCODE plugin for the Cytoscape software.

**1.4 Acquisition of hub gene**

Hub genes are generally considered to play a crucial role in the PPI network19. Cytoscape Plugin cytoHubba can sort the nodes in the PPI network by the MCC algorithm. The top ten hub genes are displayed in the main window in order of importance^(6)^. We imported the PPI network information into Cytoscape 3.8.2 software. After processing with cytoHubba, the top ten hub genes were obtained and displayed from red to yellow.

**1.5 Gene-drug interaction analysis**

Gene-drug interaction analysis can be achieved through the Drug Gene Interaction Database (DGIdb) version 4.2.0 (<https://www.dgidb.org>)^(7)^. The DGIdb database is primarily used for drug targeting and the collection of sensitive genomes and drug-gene interactions^(2)^. We used DGIdb to predict drugs or compound molecules that may interact with the top three hub genes, and the Cytoscape software was used to visualize the drug-gene interaction network.

**References**

1. Wang Q, Su G, Tan X, Deng J, Du L, Huang X, et al. UVEOGENE: An SNP database for investigations on genetic factors associated with uveitis and their relationship with other systemic autoimmune diseases. *Hum Mutat*. (2019);40(3):258-66. doi:10.1002/humu.23702.

2. Chen Z, Zhong Z, Zhang W, Su G, Yang P. Integrated Analysis of Key Pathways and Drug Targets Associated With Vogt-Koyanagi-Harada Disease. *Front Immunol*. (2020);11:587443. doi:10.3389/fimmu.2020.587443.

3. Kanehisa M, Goto S. KEGG: kyoto encyclopedia of genes and genomes. *Nucleic Acids Res*. (2000);28(1):27-30. doi:10.1093/nar/28.1.27.

4. Liu Y, Bai F, Tang Z, Liu N, Liu Q. Integrative transcriptomic, proteomic, and machine learning approach to identifying feature genes of atrial fibrillation using atrial samples from patients with valvular heart disease. *BMC Cardiovasc Disord*. (2021);21(1):52. doi:10.1186/s12872-020-01819-0.

5. Hu AL, Chan KC. Utilizing both topological and attribute information for protein complex identification in PPI networks. *IEEE/ACM Trans Comput Biol Bioinform*. (2013);10(3):780-92. doi:10.1109/TCBB.2013.37.

6. Chin CH, Chen SH, Wu HH, Ho CW, Ko MT, Lin CY. cytoHubba: identifying hub objects and sub-networks from complex interactome. *BMC Syst Biol*. (2014);8 Suppl 4:S11. doi:10.1186/1752-0509-8-S4-S11.

7. Cotto KC, Wagner AH, Feng YY, Kiwala S, Coffman AC, Spies G, et al. DGIdb 3.0: a redesign and expansion of the drug-gene interaction database. *Nucleic Acids Res*. (2018);46(D1):D1068-D73. doi:10.1093/nar/gkx1143.
