# Supplementary figures and images for: Candidate Drugs Screening for Behcet’s Disease Based on Bioinformatics Analysis and Mouse Experiments
Source: Front Immunol. 2022 Jun 21;13:895869. doi: 10.3389/fimmu.2022.895869 (PMC9253297; doi:10.3389/fimmu.2022.895869)

Supplementary Figure 1 KEGG pathway diagram of cytokine - cytokine receptor interaction

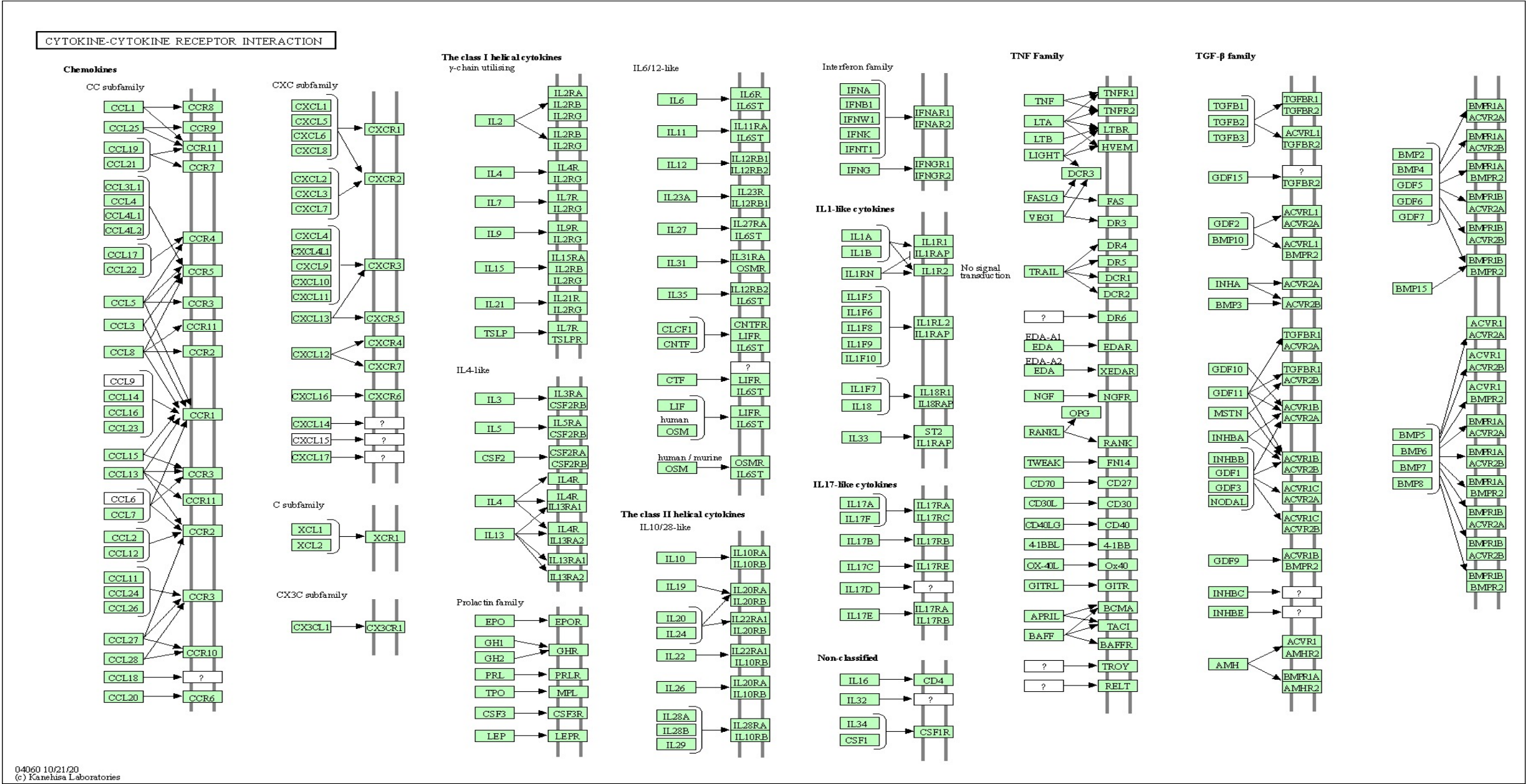

Supplement: Supplementary file 2 [file Image_1.pdf]

# Supplementary Figure 2      Inflammatory Bowel Disease pathway

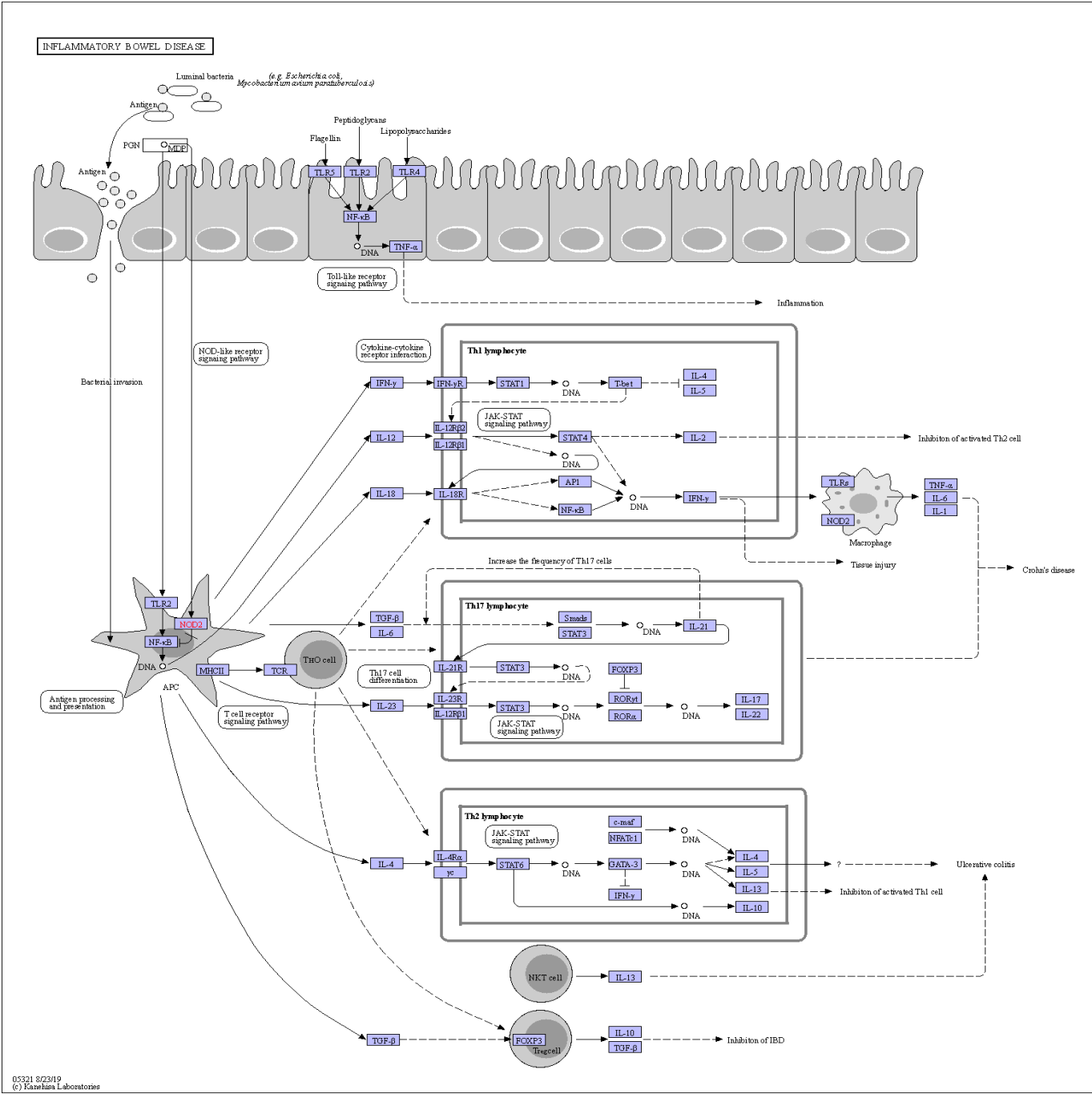

Supplement: Supplementary file 3 [file Image_2.pdf]

Supplementary Figure 3      KEGG pathway diagram of IL-17 signaling pathway

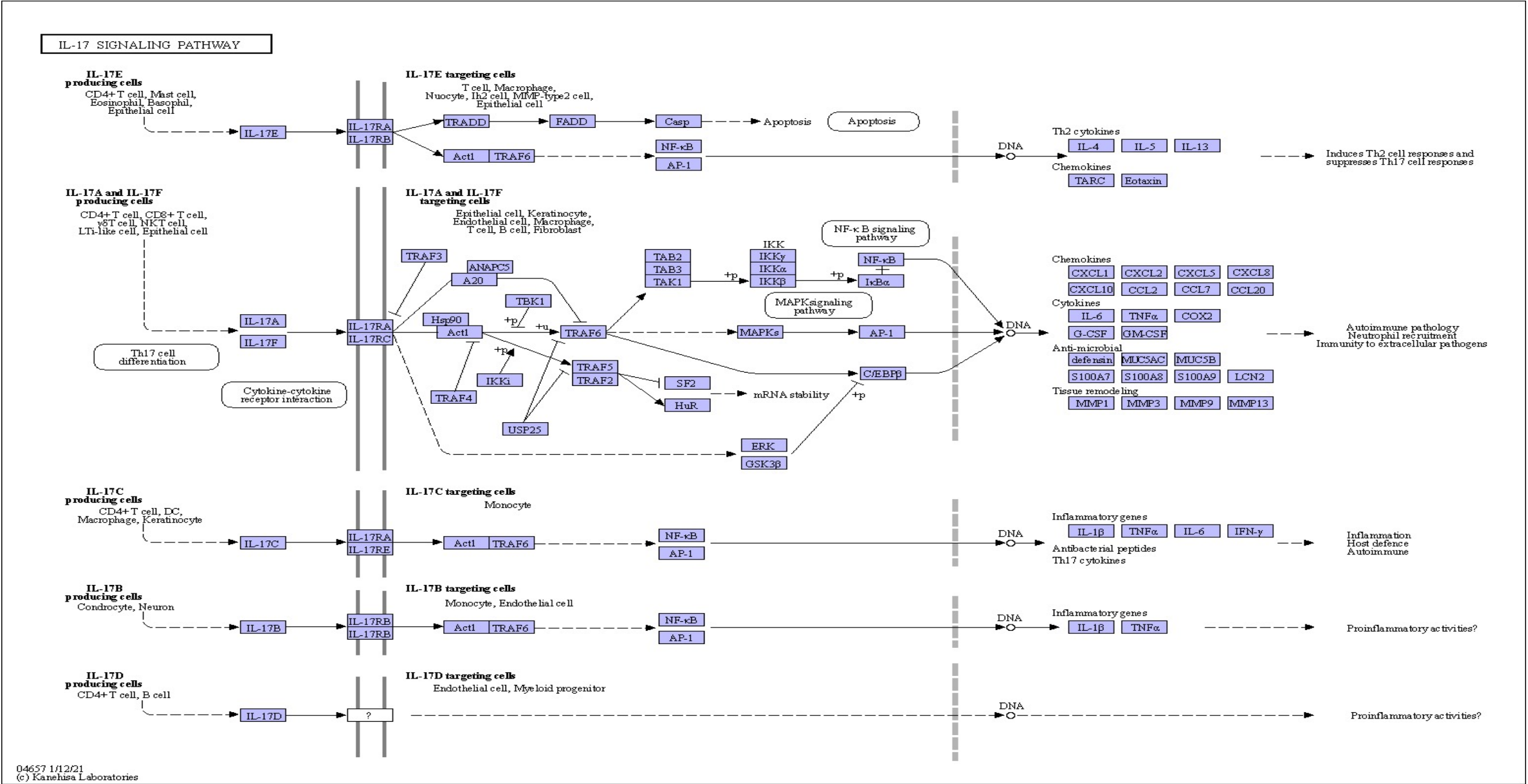

Supplement: Supplementary file 4 [file Image_3.pdf]
